# Influence of SARS-CoV-2 on Adult Human Neurogenesis

**DOI:** 10.3390/cells12020244

**Published:** 2023-01-06

**Authors:** Tomasz Stępień, Sylwia Tarka, Natalia Chmura, Michał Grzegorczyk, Albert Acewicz, Paulina Felczak, Teresa Wierzba-Bobrowicz

**Affiliations:** 1 Department of Neuropathology, Institute of Psychiatry and Neurology, 02-957 Warsaw, Poland; 2 Chair and Department of Forensic Medicine, Medical University of Warsaw, 02-007 Warsaw, Poland; 3 Department of Otorhinolaryngology, Head and Neck Surgery, Medical University of Warsaw, 02-097 Warsaw, Poland; 4 Department of Descriptive and Clinical Anatomy, Medical University of Warsaw, 00-001 Warsaw, Poland

**Keywords:** adult human neurogenesis, SARS-CoV-2, COVID-19

## Abstract

Infection with severe acute respiratory syndrome coronavirus 2 (SARS-CoV-2) is associated with the onset of neurological and psychiatric symptoms during and after the acute phase of illness. Inflammation and hypoxia induced by SARS-CoV-2 affect brain regions essential for fine motor function, learning, memory, and emotional responses. The mechanisms of these central nervous system symptoms remain largely unknown. While looking for the causes of neurological deficits, we conducted a study on how SARS-CoV-2 affects neurogenesis. In this study, we compared a control group with a group of patients diagnosed with COVID-19. Analysis of the expression of neurogenesis markers showed a decrease in the density of neuronal progenitor cells and newborn neurons in the SARS-CoV-2 group. Analysis of COVID-19 patients revealed increased microglial activation compared with the control group. The unfavorable effect of the inflammatory process in the brain associated with COVID-19 disease increases the concentration of cytokines that negatively affect adult human neurogenesis.

## 1. Introduction

For a few years, people have been struggling with the COVID-19 pandemic caused by severe acute respiratory syndrome coronavirus 2 (SARS-CoV-2) [1]. Subsequent mutations have led to COVID-19 waves of infection with significantly varying symptoms, prognoses, and transmissibility levels. Reports to date are mainly associated with coronavirus structural proteins and, in particular, the receptor binding domains of spike (S)-proteins rather than other nonstructural and accessory proteins [2]. SARS-CoV-2 B.1.617.2, also termed the Delta variant, was classified as a variant of concern according to the World Health Organization (WHO), demonstrating both increased transmissibility and increased disease severity. In contrast, the Omicron (B.1.1.529) variant seemed more infectious than the Delta variant and more likely to lead to vaccine breakthrough infections; it has also been reported to induce milder symptoms in most patients [3].

WHO statistics show that more than 628 million people have been infected with SARS-CoV-2 and approximately 6.6 million have died; however, the true numbers are significantly higher (see https://covid19.who.int, https://ourworldindata.org, accessed on 2 December 2022). The coronavirus disease pandemic continues due to the spread of new variants of SARS-CoV-2 worldwide [4,5]. The typical symptoms of COVID-19 include anosmia and ageusia along with fever, dry cough, and shortness of breath [6,7]. COVID-19 is a neurotropic virus associated with neurological manifestations in up to 36% of patients [8], and the most commonly reported manifestations are cerebrovascular events, followed by altered mental status [9]. Neurological manifestations can range from a mild headache or “brain fog” [10] to more serious complications, such as Guillain-Barre syndrome [11], encephalitis [12], and arterial and venous strokes [13]. The SARS-CoV-2 vaccine is still under development, and there is no specific drug at present. Many antiviral drugs have been employed for the treatment of SARS-CoV-2 infection, but have not been effective yet. Prompt development of effective drugs for COVID-19 therapy is a difficult task as the conventional drug development process usually takes a long time and costs billions. A comparative genomics-based approach with earlier known human CoVs can provide a breakthrough in COVID-19 therapeutics [14,15,16].

SARS-CoV-2 enters the human body via the angiotensin-converting enzyme (ACE)-2 receptor, which has been found to be expressed by airway epithelia, lungs, choroid plexus and various brain cells, including endothelial cells of the cerebral microvascular system [17,18]. Angiotensin-converting enzyme 2 (ACE2)- and transmembrane serine protease 2 (TMPRSS2)-expressing ciliated cells of the nasal mucosa are the primary targets of initial SARS-CoV-2 infection [19]. Infection with SARS-CoV-2 primarily leads to respiratory tract infection, and its sequelae frequently dominate the clinical course [20]. The olfactory tract seems to be the principal entry route to the CNS in the initial phases of SARS-CoV-2 infection [21]. Moreover, a high fraction of patients, perhaps as high as 33%, continue to suffer neuropsychiatric symptoms posthospital discharge, including a dysexecutive syndrome consisting of inattention, disorientation, and poor movement coordination [22,23,24,25].

Postmortem human neuropathological findings in COVID-19 include hypoxic damage, microglial activation, astrogliosis, leukocytic infiltration, and microhemorrhages, suggesting that, at least in some cases, the CNS undergoes neuropathological sequelae associated with hypoxia and neuroinflammation [26,27]. This is supported by neuroimaging studies in postacute COVID-19 patients, showing disruption of fractional anisotropy and diffusivity, suggesting microstructural and functional alterations of the hippocampus, a brain region critical for memory formation, and part of a conserved subcortical network involved in anxiety and stress responses. Thus far, the neurobiological bases of COVID-19 neuropsychiatric symptoms remain largely unknown [28]. Disruption of the blood–brain barrier (BBB) and damage to tight junctions may occur during COVID-19 infection. The BBB is crucial in protecting the hemodynamic function of the brain. The interconnected nature of brain capillary endothelial cells, pericytes, neurons, astrocytes, and microglia in the BBB strongly suggests this to be a path of SARS-CoV-2 viral entry to the brain and a contribution to neuroinflammatory events [29]. Evidence from in vitro models has shown that isolated spike proteins can cross the BBB [30]. While all regions examined, including the olfactory bulb (OB), cortex, hippocampus, and medulla oblongata, showed some degree of BBB disruption, the hippocampus suffered the most significant changes [31,32]. Recent studies have shown that people with COVID-19 are at a significantly increased risk of a new diagnosis of Alzheimer’s disease within 360 days of the initial COVID-19 diagnosis, especially those aged ≥ 85 years [33].

The hippocampus is one of the two regions where new neurons are generated. In the adult human brain, two locations of adult human neurogenesis, the dentate gyrus (DG) and the subventricular zone (SVZ), have been identified. Therefore, we wanted to explore how SARS-CoV-2 influences adult human neurogenesis. Neurogenesis involves the proliferation and differentiation of progenitor cells, as well as the migration and maturation of newly formed neurons. The first direct evidence for the occurrence of neurogenic processes in the adult human brain was described in 1998 by Erickson [34]. He revealed the generation of new neurons in the dentate gyrus and subventricular zone [35]. Newborn neurons migrate from the SVZ toward the olfactory bulb (OB). The fate of newborn neurons in the DG is strictly determined topographically, and they do not show a tendency to migrate toward the brain’s neocortical areas. They only participate in the constant supplementation of the pool of new granule cells in the dentate. In the DG, three types of transcriptionally active cells were identified: neural stem cells glia-like (NSCs, type-I cells), cells without processes (NSCs, progenitor cells, type-II cells), and neuroblasts [36]. In the SVZ, three types of transcriptionally active cells have been distinguished, namely, GFAP-positive neural stem cells (NSCs, type-B1 cells), progenitor cells (NSCs, type-C cells), and neuroblasts (type-A cells) [37]. Markers of early adult human neurogenesis phases comprise DCX (microtubule-associated protein expressed during neuronal migration) and NeuN (neuronal nuclear antigen), which label migrating neuroblasts and immature neurons, as well as GFAP (glial fibrillary antigen protein), which labels astrocytic stem cells [38,39]. Phosphorylated histone H3Ser-10 (p-Histone H3Ser-10) is more precise, and contrary to DCX, its expression occurs only in newborn neurons (neuronal progenitor cells, NPCs) [40]. Phosphorylation of the N-terminal domain of histone H3 at position Ser-10 and/or Ser-28 destabilizes chromatin, directly preceding replication and transcription [41,42].

Adult human neurogenesis is regulated by endogenous and exogenous factors that influence the proliferation potential of progenitor cells and accelerate the rate of development of the dendritic connections of newly formed neurons. The factors influencing the dynamics of neurogenesis and the total number of neurons include stress, diet, physical activity, alcohol, drugs, and medications [43]. Increased levels of proinflammatory factors may contribute to the formation and development of newly formed neurons. Proneurogenic importance during inflammation is shown by proteins secreted by active microglia, mainly CD47 and CD55 and interleukins 4 and 10. Analysis of COVID-19 patients revealed increased microglial activation and IL-1β expression compared with the control group. Elevated expression of IL-6 was detected in neurons, suggesting neuronal cytokine production [44]. However, the brain inflammation associated with COVID-19 increases the concentration of cytokines that negatively affect adult human neurogenesis, e.g., IL-6, IL-1β, IL-1α, and TNF [45]. Among the factors that reduce neurogenesis, the factor that almost completely suppresses neurogenesis is Zika virus (ZIKV). The CNS developmental arrest observed in congenital Zika syndrome is beyond neuronal cell death. Interestingly, Zika virus, as one of the proapoptotic factors, is very similar to SARS-CoV-2 and belongs to the same group of nonsegmented positive-sense RNA. Some studies have reported a decrease in neurogenesis in patients with COVID-19, linking it with brain fog and other neurological symptoms [10].

We hypothesized that the inflammatory response present in COVID-19 changes adult human neurogenesis. The aim of our study was to identify neuronal progenitor cells (NPCs) and perform a quantitative analysis of neurogenesis cell density, as well as to compare the group of patients with SARS-CoV-2 and the control group.

## 2. Materials and Methods

The study material was derived from the Brain Bank at the Institute of Psychiatry and Neurology, Warsaw, Poland. It comprises two structures, the hippocampal dentate gyrus and the subventricular zone. All brains were fixed in buffered 4% formaldehyde and embedded in paraffin.

The whole study group consisted of 52 patients with many different comorbidities. The final study group was composed of brains derived from 14 patients (7 men, 7 women, mean age 62.5 ± 6.9 years) with a COVID-19 diagnosis. The criteria for inclusion in this study were a positive result for the nasopharyngeal swab for SARS-CoV-2 RNA (PCR), pathological/radiological diagnosis of pneumonia of SARS-CoV-2, and/or presence of SARS-CoV-2 nucleoprotein in pulmonary tissue confirmed by immunohistochemical examination (IHC). The exclusion criteria in this study were hemorrhage, ischemic stroke, and cancer. Half of the study group had no comorbidities, and the rest had hypertension, chronic obstructive pulmonary disease (COPD), ischemic heart disease, and diabetes. Brains from the control group had no significant neuropathological lesions. They were derived from 8 patients (6 men, 2 women, mean age 64 ± 10.95 years) whose deaths occurred sporadically (within less than 10 min) without comorbidities and did not meet the study inclusion criteria. The tissue samples embedded in paraffin were cut into 5 µm slices and stained with hematoxylin and eosin and Mallory trichrome. Moreover, the study material was analyzed by IHC with the p-Histone antibodies H3_Ser-10_ (Proteintech, 66863-1-IG,1:2200, Rosemont, IL, USA), LCA (Dako, 8B11-PD7/26, 1:75, Los Angeles, CA, USA), and NeuN (Millipore MAB377, 1:100, Darmstadt, Germany).

### Quantitative Analysis

Neuronal progenitor cells were counted in the hippocampal dentate gyrus (DG) and subventricular zone (SVZ) of the lateral ventricles. The quantitative analysis was performed by CellSens software (Tokyo, Japan). The results were analyzed with STATISTICA 12 (TIBCO Software Inc., Poland) software.

## 3. Results

**Figure 1:**
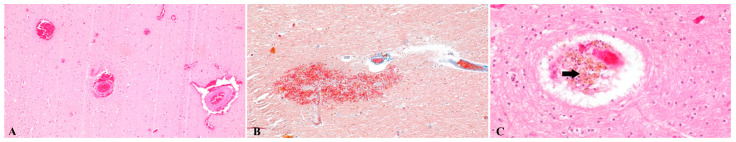
Group patients with diagnosed COVID-19. Temporal lobe. Microbleeds/petechial hemorrhages around the vessels. (**A**). Hematoxylin and eosin stain (H&E). Magnification ×100. (**B**). Mallory trichrome stain. Magnification ×200. (**C**). Frontal lobe. Hemosiderophages (arrow). Hematoxylin and eosin stain (H&E). Magnification ×400.

We analyzed the experimental material in terms of neuropathological changes due to COVID-19. In all patients who died in the course of COVID-19, we observed perivascular changes, microbleeding/petechial hemorrhages, and hemosiderophages (Figure 1).

**Figure 2:**
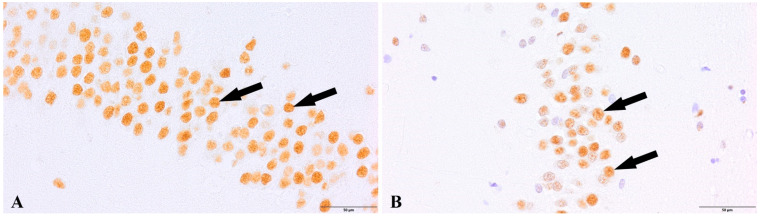
Dentate gyrus, neural progenitor cells (NPCs, arrows). (**A**). Control group. (**B**). Group patients with diagnosed COVID-19. Immunolabeling p-Histone H3Ser10 antibody.

**Figure 3:**
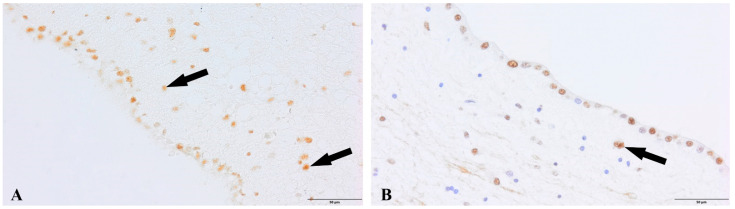
Subventricular zone, neural progenitor cells (NPCs, arrows). (**A**). Control group. (**B**). Group patients with diagnosed COVID-19. Immunolabeling p-Histone H3Ser10 antibody.

**Figure 4:**
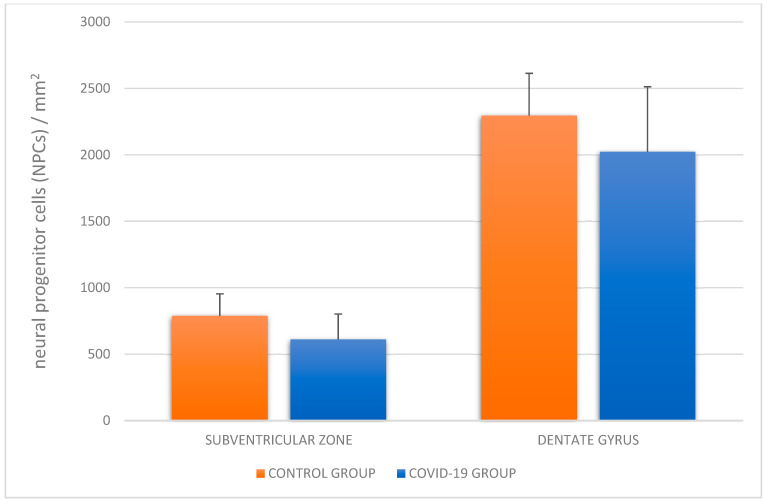
Density of neural progenitor cells (NPCs) in the hippocampal dentate gyrus and subventricular zone in the control group and group of patients with diagnosed COVID-19.

We observed a positive immunohistochemical response in transcriptionally active nuclei of the neuronal progenitor cells in the hippocampal dentate gyrus (DG) (Figure 2) and subventricular zone (SVZ) of both experimental groups (Figure 3). In the study group of patients diagnosed with COVID-19, we observed a decreased density of neuronal progenitor cells in the DG and SVZ. Quantitative analysis of neuronal progenitor cells (NPCs) with p-H3_Ser-10_ expression in the subventricular zone (610.9 ± 164.7 NPCs/mm^2^) and dentate gyrus (2024.1 ± 190.9 NPCs/mm^2^) revealed decreased density in the group of patients with SARS-CoV-2 compared to the control group (DG 2295.5 ± 488.5 NPCs/mm^2^; SVZ 788.8 ± 318.5 NPCs/mm^2^) (Figure 4). The statistical analysis showed no significant differences between the group of patients with diagnosed COVID-19 and the control group.

**Figure 5:**
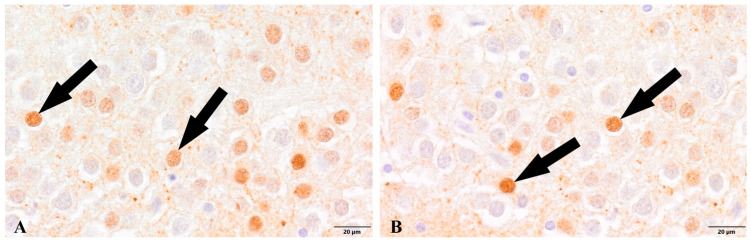
Dentate gyrus, newborn neurons (arrows). (**A**). Control group. (**B**). Group patients with diagnosed COVID-19. Immunolabeling NeuN antibody.

**Figure 6:**
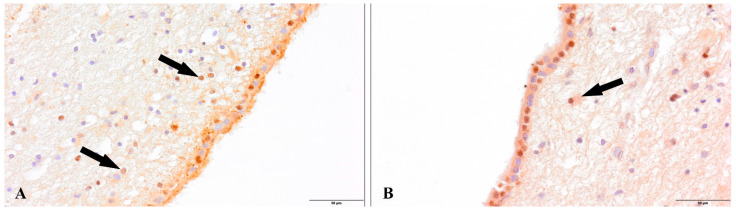
Subventricular zone, newborn neurons (arrows). (**A**). Control group. (**B**). Group patients with diagnosed COVID-19. Immunolabeling NeuN antibody.

In both experimental groups, we observed the presence of newborn, maturing neurons labeled with NeuN (Figure 5 and Figure 6). Maturing neurons were not quantified, while the analysis of selected cases showed a reduced density of newborn neurons in the group of patients with diagnosed COVID-19 in the hippocampal dentate gyrus.

**Figure 7:**
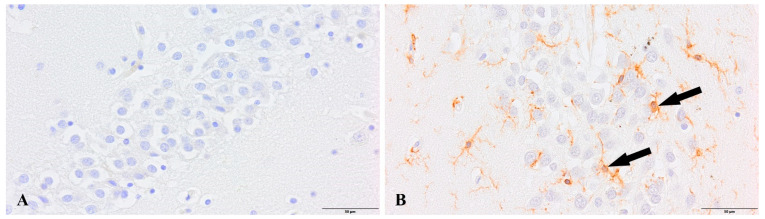
Dentate gyrus. (**A**). Control group. (**B**). Active ramified microglia (arrows). Group patients with diagnosed COVID-19. Immunolabeling LCA antibody.

**Figure 8:**
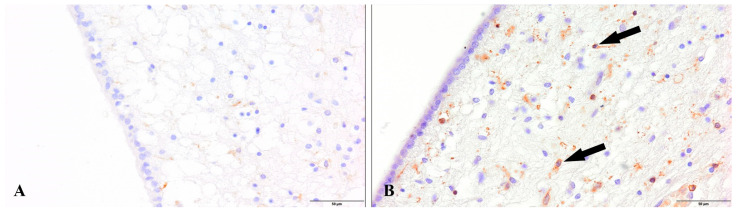
Subventricular zone. (**A**). Control group. (**B**). Active ramified microglia (arrows). Group patients with diagnosed COVID-19. Immunolabeling LCA antibody.

To mechanistically dissect the reasons for the decreased density of neuronal progenitor cells, we performed labeling with LCA antibodies. We observed the presence of active, ramified microglia in the hippocampal dentate gyrus (Figure 7) and subventricular zone (Figure 8) only in the group of patients with diagnosed COVID-19.

## 4. Discussion

The neuropathological changes observed in the COVID-19 group included the subarachnoid space and around the blood vessels of the parenchyma, microbleeds/petechial hemorrhages, and hemosiderin deposits, suggesting previous petechial hemorrhage. Other neuropathological changes have also been described in the available literature. Postmortem human neuropathological findings in COVID-19 include hypoxic damage, microglial activation, astrogliosis, leukocytic infiltration, and microhemorrhages, suggesting that, at least in some cases, the CNS undergoes neuropathological sequelae associated with hypoxia and neuroinflammation [26,27,46].

The hippocampus and subventricular zone are key neurogenic areas of the adult brain that harbor neural stem cells. The hippocampus is one of the important functional regions of the limbic system of the brain that contributes to the neuroregenerative process, long-term potentiation, learning process, memory formation, and regulation of emotion. In the hippocampus of a healthy adult, approximately 700 new neurons are generated every day [47]. Defects in hippocampal structure and functions due to aging and neurological illnesses have been directly linked to emotional disorders and memory loss [28]. Impaired neurogenesis has been identified as a potential cause of cognitive decline and progressive memory loss in aging and neurodegenerative diseases, particularly Alzheimer’s disease, Parkinson’s disease, and Huntington’s disease [48,49,50]. Various neurocognitive disturbances in COVID-19 patients suggest the localization of lesions within the hippocampus. This was confirmed by neuroimaging (magnetic resonance imaging (MRI)) in patients after acute COVID-19 and microstructural and clinical studies of hippocampal changes [28].

The results of our research showed that SARS-CoV-2 could disrupt the process of neurogenesis. We observed a decrease in the density of neural progenitor cells in the hippocampal dentate gyrus and subventricular zone in the group of patients diagnosed with COVID-19 compared to the control group. Our observations of some cases also suggested a lower density of newborn neurons in the dentate gyrus of the hippocampus. The exclusion criteria were restrictive to exclude the influence of other factors on neurogenesis. There is experimental evidence that SARS-CoV-2 is able to infect neural progenitor cells, leading to impaired neuroblast maturation and neuronal death [51,52,53].

One of the possible answers to the question of what causes the disturbance of neurogenesis may be proinflammatory cytokines and the presence of active microglia in the hippocampal dentate gyrus and subventricular zone. This was confirmed by our study. In the group of patients diagnosed with COVID-19, we observed the presence of active ramified microglia in both the DG of the hippocampus and SVZ.

Upon infection, SARS-CoV-2 replicates in tissues and organs and induces peripheral and local cytokine storms that potentially deteriorate the innate immune system. Based on the experimental data derived from immunological assays in the plasma samples of COVID-19 patients, elevated levels of key proinflammatory determinants, including different interleukins (ILs), fibroblast growth factor (FGF), interferon-gamma (IFN-γ), tumor necrosis factor-alpha (TNF-α), and vascular endothelial growth factor (VEGF), have become evident. Among them, the surplus levels of IFN-γ, TNF-α, IL-1, and IL-6 have been known to be associated with the dysfunction of the blood–brain barrier (BBB) as a part of priming the neuroinflammatory process in the human brain during COVID-19 [32,54,55,56].

Moreover, neuropathological studies indicating vascular pathology and hypoxic neuronal damage in the hippocampus of COVID-19 patients correlate with emerging dementia [26]. Our results may explain the frequent presence of brain fog in the course of COVID-19, which is associated with increased hypoxia.

ZIKV, which is one of the few factors that almost completely silences neurogenesis, operates in a similar mechanism. ZIKV, such as SARS-CoV-2, is a positive-sense RNA virus. Zika virus has been shown to directly infect neuronal progenitor cells, leading to apoptosis [57]. Receptor NPC IL-1β is involved in reducing neurogenesis in murine models of brain inflammation [58,59].

Long COVID-19 and post-COVID-19 changes and their impact on adult human neurogenesis remain open questions. The increasing death rate from COVID-19 around the world is a serious problem, but a significant proportion of COVID-19 survivors appear to be at increased risk of various neurological deficits. Several studies have shown a decrease in the level of neurogenesis and the associated memory impairment caused by elevated levels of stress hormones and proinflammatory factors in the brain, which disrupt the neuroplasticity of the hippocampus and could be a potential cause of dementia in a significant proportion of patients with a history of COVID-19. It has been suggested that the probability of replenishing dysfunctional and degenerated neurons is then the lowest. We do not know how long elevated markers of inflammation persist in survivors. The persistence of neuropsychiatric symptoms in chronic COVID-19 suggests that neuronal damage may be prolonged. Animal model studies suggest that the inflammatory changes are transient, decreasing after removal of the virus from the nasal cavity [56].

The changes in neuronal progenitor cells induced by ZIKV are different from those induced by SARS-CoV-2, and their effects appear to be reversible in SARS-CoV-2. Due to the recurring waves of the pandemic, there is an urgent need for a detailed analysis of the misfire of hippocampal neurogenesis due to cognitive impairment in COVID-19.

## 5. Limitations

The present study’s strengths include a relatively large group of patients with a COVID-19 diagnosis. However, limitations should be acknowledged. Perhaps factors other than SARS-CoV-2, which we do not know, had an impact on the reduction of neurogenesis. However, patients in both groups were free of comorbidities or had comorbidities not affecting neurogenesis.

## Data Availability

https://covid19.who.int (accessed on 2 December 2022). https://ourworldindata.org (accessed on 2 December 2022).
